# Vitamin D and Postpartum Depression: A Narrative Review

**DOI:** 10.3390/clinpract16080147

**Published:** 2026-08-08

**Authors:** Afra Almashghouni, Haydar Hasan, Dimitrios Papandreou

**Affiliations:** Department of Clinical Nutrition and Dietetics, College of Health Sciences, University of Sharjah, Sharjah 27272, United Arab Emirates; almashghouni.92@gmail.com (A.A.); haidarah@sharjah.ac.ae (H.H.)

**Keywords:** vitamin D, postpartum depression, 25-hydroxyvitamin D, serotonin, neuroinflammation, maternal mental health, perinatal depression

## Abstract

**Background/objective:** Postpartum depression (PPD) is a significant public health issue affecting about 19% of mothers globally. It has well-documented impacts on maternal well-being, mother–infant relationships, and child developmental outcomes. At the same time, the level of vitamin D deficiency in women of reproductive age is very high (affecting 60–87 percent of pregnant women globally). The review aims to synthesize current evidence and highlight priority research areas to improve maternal mental health outcomes. **Methods:** A systematic literature search of PubMed, Scopus, and Google Scholar (2015–2026) was conducted, followed by narrative synthesis of mechanistic, observational, and interventional evidence. **Results:** Adequate vitamin D status may contribute to a reduced risk of postpartum depression through multiple interacting pathways, including serotonin synthesis and metabolism (via tryptophan hydroxylase 2 (*TPH2*) and monoamine oxidase-A (*MAO-A*) regulation), neuroplasticity via brain-derived neurotrophic factor (*BDNF*) signaling, and suppression of the pro-inflammatory cytokine cascade. Dose–response meta-analyses and large prospective cohort studies indicate consistent negative relationships between maternal vitamin D levels and postpartum depressive and anxiety symptoms with optimal serum 25-hydroxyvitamin D (25(OH)D) levels of 90–110 nmol/L. **Conclusions:** There is still limited evidence on the intervention; it is still methodologically diverse, and only a few randomized controlled trials have been carried out on specifically postpartum populations.

## 1. Introduction

Depression ranks as one of the leading causes of worldwide disability and is a significant public health concern across all stages of life. The prevalence of depression is about 2 times higher in women than in men, and the inequality is more pronounced in the reproductive years [1]. Postpartum depression (PPD) is the most frequent complication of childbirth among the depressive conditions that affect women, with 12–19% of mothers being globally affected [1]. PPD is characterized by persistent sadness, emotional dysregulation, sleep disturbance, anhedonia, and in some cases, intrusive thoughts. It typically occurs during the first year postpartum and may persist for months or years without treatment. The impact of PPD is not just restricted to the mother: it is associated with disrupted mother-infant bonds, disrupted early attachment, poor child development in cognitive and behavioral aspects, and extreme partner and family stress [1]. Despite the growing awareness and more frequent screening that is now widespread in most healthcare systems, PPD remains grossly underdiagnosed and undertreated, which brings to the fore the need to determine the modifiable biological and dietary risk factors.

Over the past few years, there has been a significant body of research on vitamin D as a potentially influential modulator of mental health. Though traditionally related to calcium homeostasis and bone metabolism, it is now clearly known that vitamin D is a steroid hormone that has extensive systemic effects, including those in the central nervous system (CNS) [2,3]. Vitamin D receptors (*VDR*) and the enzyme 1-alpha-hydroxylase, which converts vitamin D to its active form locally, are found throughout the areas of the brain that are essential to mood regulation, such as the limbic system, hippocampus, and prefrontal cortex [2]. Such neuroanatomy in the body gives a strong biological foundation for the possible effect of vitamin D in the pathophysiology of depression [2,3,4].

Vitamin D deficiency is also one of the most common micronutrient deficiencies on the planet [5]. The combination of reduced exposure to sunlight, inadequate dietary intake, heightened physiological demand during pregnancy and lactation, coupled with cultural or environmental factors such as diminished exposure to ultraviolet B (UVB), makes women of childbearing age particularly susceptible [5]. Population statistics show that 60–87 percent of pregnant women have serum 25-hydroxyvitamin D [25(OH)D] concentrations that are below the recommended levels, with significant geographic, seasonal, and skin pigmentation differences [5]. The greatest prevalence of deficiency is in Sub-Saharan African and South-East Asian populations, with the average levels of maternal 25(OH)D between 13 and 52 nmol/L in certain areas [5].

Meta-analyses suggest that vitamin D supplementation is associated with reductions in depressive symptoms, particularly in deficient patients, though results are heterogeneous [3,6,7,8,9]. These discrepancies underscore the need to take into consideration biological context, life stage, and baseline nutritional status during the assessment of the possible mental health impact of vitamin D.

A unique biological environment created by the inflammatory influx, hormone changes, and immune functional alterations that characterize pregnancy and the immediate postpartum has the potential to connect vitamin D status in a meaningful manner [10,11]. Epidemiological and prospective research has found that low levels of vitamin D in pregnancy or at birth lead to high PPD rates [12,13,14,15,16]. Interventional studies have reported that maternal vitamin D deficiency (less than 50 nmoI/L) relates to a significantly high occurrence of PPD [13,14,17]. Even though observational studies cannot determine causality, the likelihood that the temporal sequence observed in cohort studies is true is that the sequence is prospective, so a plausible causal pathway should be considered carefully. RCTs in this particular population are still scarce and heterogeneous in methodology [12].

In the present review, vitamin D deficiency was defined as serum 25-hydroxyvitamin D [25(OH)D] concentrations < 50 nmol/L, consistent with recommendations from the Institute of Medicine (IOM) and criteria commonly used in pregnancy research. Although some investigators have adopted lower thresholds (<30 nmol/L) to define deficiency, the 50 nmol/L cutoff was selected because it is the most widely accepted threshold for identifying inadequate vitamin D status and has been used in several studies examining PPD risk. Nevertheless, the optimal threshold remains controversial, and differences in diagnostic criteria may contribute to inconsistencies across studies.

Although randomized controlled trials (RCTs) investigating vitamin D supplementation during pregnancy for the prevention or treatment of PPD are limited, they are not entirely absent. A small number of RCTs have reported reductions in depressive symptoms among women receiving vitamin D supplementation; however, these studies were generally characterized by small sample sizes and methodological heterogeneity, preventing definitive conclusions regarding efficacy [12]. Consequently, further large-scale, well-designed prospective trials are needed to establish a causal relationship between maternal vitamin D status and PPD.

There are occasional lines of evidence that have enhanced the biological argument on the role of vitamin D in mental health [18,19,20,21]. Mechanistic studies have proposed various pathways linking vitamin D to mood regulation, including calcium signaling [22], gut–brain interactions [23] and broader nutrient deficiency frameworks [24].

This narrative review considers the connection between vitamin D status and PPD by synthesizing three areas that are interconnected: first, the biological processes by which vitamin D might affect mood, including immune, hormonal, and neurotransmitter pathways; second, biomarker and nutritional evidence about vitamin D in the postpartum period; and third, observational and interventional studies specific to postpartum populations. The review will aim to elaborate on what the current supporting evidence is, areas of incongruity, and the most important areas of research that should be conducted in order to improve maternal mental health outcomes. While the primary focus of this review is PPD, mechanistic evidence is drawn from preclinical studies, general population research, and other psychiatric conditions where PPD-specific data are unavailable. Wherever such extrapolated evidence is presented, we explicitly note its limitations and the need for PPD-specific confirmation.

## 2. Materials and Methods

The literature search involved the use of PubMed, Scopus, and Google Scholar to find the publications discussing vitamin D status, dietary intake, or supplementation in terms of depression and PPD. To include the latest evidence, the search was limited to the last 10 years (2015–2026). This narrative review was completed as per the PRISMA (Preferred Reporting Items for Systematic Reviews and Meta-Analyses) on Narrative Reviews to guarantee transparency in the process of search, selection, and reporting. We adopted this approach: a systematic search with narrative synthesis to comprehensively identify relevant literature while acknowledging that formal meta-analytic pooling was not appropriate given the heterogeneity of study designs, populations, and outcome measures across the identified studies. This methodological choice allows us to provide a comprehensive overview of the current evidence base while transparently presenting the search and selection process. Three main concepts were used to develop search strategies: the exposure (vitamin D), the outcome (depression or PPD), and the study design. They were operated with the help of Boolean operators: OR within the concept groups and AND to connect concepts. Exposure terms included ‘vitamin D’, ‘25-hydroxyvitamin D’, ‘25(OH)D’, ‘cholecalciferol’, ‘ergocalciferol’, ‘1,25-dihydroxyvitamin D’, ‘calcifediol’, ‘calcitriol’, and ‘supplementation’. Outcome terms comprised ‘postpartum depression’, ‘postpartum depressive symptoms’, ‘postnatal depression’, ‘perinatal depression’, and ‘maternal depression’. The study design filters were randomized controlled trials, cohort studies, case–control studies, cross-sectional studies, systematic reviews, and meta-analyses.

**Figure 1:**
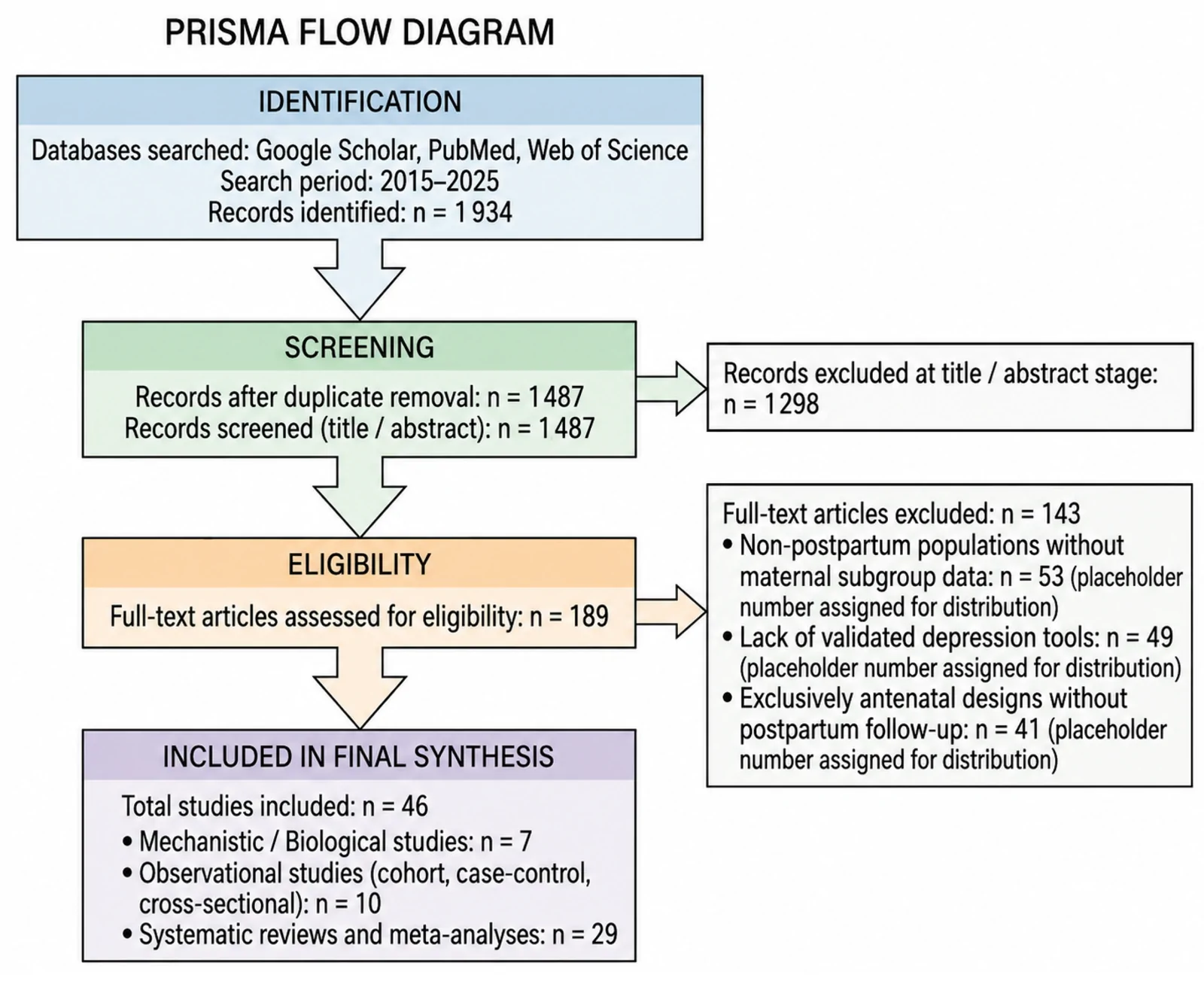
PRISMA-style flow diagram documenting the systematic literature search and study selection process for this narrative review. The diagram is presented for transparency in reporting search methodology, consistent with PRISMA guidelines for systematic searching in narrative reviews.

Record screening was carried out in two stages. Three databases (Google Scholar, PubMed, Web of Science) were searched and revealed 1934 records. After eliminating duplicates, 1487 records were vetted at the title and abstract stage, where 1298 were eliminated as irrelevant. Table 1 summarizes the criteria used to screen studies for inclusion in this review. The narrative synthesis included a final sample of 46 studies that included: 7 mechanistic or biological studies, 10 observational studies (cohort, case–control, and cross-sectional design), and 29 systematic reviews and meta-analyses. The PRISMA-style flow diagram (Figure 1) shows this search process. Table 2A–C gives a summary of all 46 studies included. The methodological limitations inherent in narrative synthesis, including reliance on extrapolated mechanistic evidence and the absence of formal quality assessment, should be considered when interpreting these findings.

The papers were then evaluated according to the eligibility criteria (Table 1) after the elimination of duplicates. The inclusion criteria included the presence of human subjects, the measurement of vitamin D status or intake, and the description of the outcomes of depression with a validated assessment instrument. A reference management was conducted with Zotero (version 6.0; Corporation for Digital Scholarship, Vienna, VA, USA) to organize, de-duplicate, and format citations.

It is important to note that the mechanistic evidence presented in the next Section 3.1 is derived primarily from:

- Preclinical (animal and cell culture) studies
- General population research
- Other psychiatric conditions (PTSD, Alzheimer’s disease, post-stroke depression)

These studies provide biological plausibility for the vitamin D-PPD association but require confirmation in postpartum populations. PPD-specific evidence, where available, is presented in Section 3.2. We have explicitly noted evidence sources throughout to help readers distinguish between established PPD-specific findings and extrapolated mechanistic evidence.

## 3. Results

### 3.1. Mechanisms Linking Vitamin D to Depression

Vitamin D deficiency and depression are complex and multi-level biological pathways that interact in a complex manner to affect the functioning of the brain, emotional regulation, and stress resilience [2,3,4,5,6,7,8,25]. Preclinical research, human neuroimaging studies, and epidemiological studies have converged in the last ten years to describe a few likely mechanisms. The comprehension of these pathways is not only essential to understanding the pathophysiology of depression but also to defining possible therapeutic targets and guiding the use of supplementation. In Table 3, the main mechanisms mentioned in this section are summarized.

**Figure 2:**
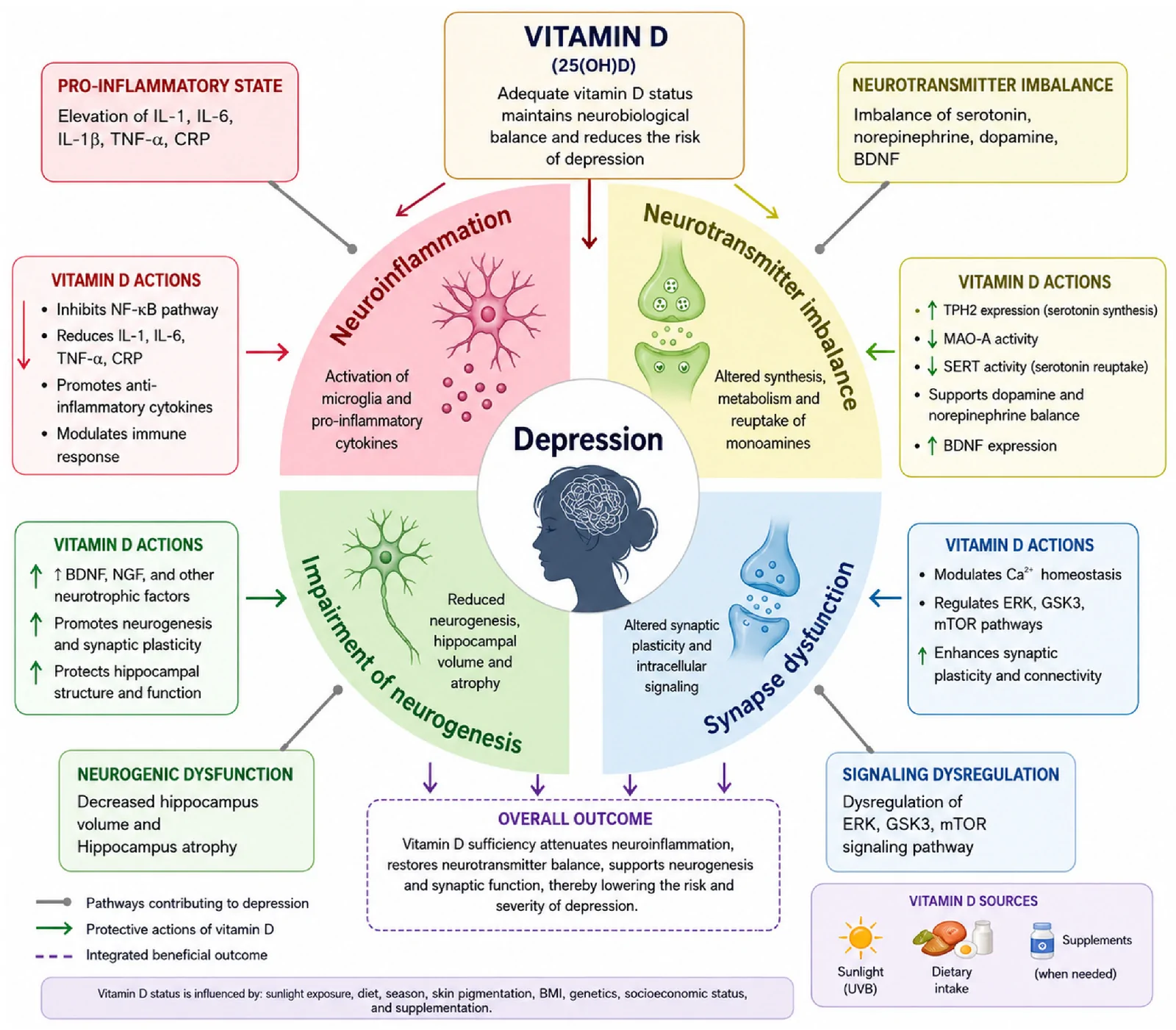
An AI-Generated Diagram Summarizing Vitamin D’s Neurobiological Mechanisms Relevant to depression.

After cutaneous synthesis or dietary intake, vitamin D_3_ binds to vitamin D binding protein (VDBP) and is transported to the liver where it is hydroxylated to 25(OH)D, the primary circulating form and the standard clinical assessment of vitamin D status. 25(OH)D is further hydroxylated in the kidneys as well as locally in the brain tissue to the biologically active form, 1,25-dihydroxyvitamin D_3_ (calcitriol), which binds the nuclear *VDR* to mediate gene transcription [2,7]. The presence of *VDR* and 1-alpha-hydroxylase in mood-regulating brain regions including the prefrontal cortex, hippocampus, cingulate cortex, and hypothalamus confirms that vitamin D’s neurobiological effects are not merely peripheral effects [2,3,37]. Notably, genetic research has confirmed that vitamin D signaling in the neurodevelopmental processes that are critical to mood regulation: genome-wide association studies have detected common loci between vitamin D metabolism and mood-related disorders (including genes whose expression patterns have lifespan-specific differences, such as *TRMT61A*, *ITIH4*, *RASGRP1*, *GRM5* [21]. Figure 2 depicts the interconnected mechanisms by which vitamin D has its effects on the neurobiological mechanisms of depression, including serotonergic neurotransmission, neuroinflammation, HPA axis regulation, neurotrophic support, and oxidative stress defense.

#### 3.1.1. Serotonin and Neurotransmitter Regulation

The evidence presented in this section is derived primarily from preclinical studies (rat serotonergic neuronal cells), general population research, and mechanistic studies. Direct PPD-specific evidence for these mechanisms is limited, and the relevance to postpartum populations requires confirmation through perinatal-focused research.

Vitamin D is a key player in the synthesis and metabolism of serotonin, the most heavily implicated monoamine neurotransmitter in mood disorders [2,4,7]. The active form, 1,25-dihydroxyvitamin D_3_, is a secosteroid hormone in the CNS by binding to VDRs located in regions of the prefrontal cortex, hippocampus, and hypothalamus [3,4,5]. These ligand-activated transcription factors control the expression of many genes, including those that control the synthesis and metabolism of neurotransmitters [2,7]. *VDR* activation upregulates tryptophan hydroxylase 2 (*TPH2*) via a vitamin D response element (VDRE) in the *TPH2* promoter, thereby increasing serotonin biosynthetic capacity [2,4,7]. Concurrently, 1,25(OH)_2_D_3_ inhibits serotonin transporter (SERT) activity and suppresses monoamine oxidase-A (*MAO-A*), the primary enzyme of serotonin degradation [1,2,7,37].This combined regulation of synthesis, reuptake, and degradation suggests that vitamin D insufficiency may impair serotonergic function across multiple nodes, potentially contributing to depressive symptomatology—a pattern consistent with observed inverse associations between serum 25(OH)D and depression risk [3,6,7,8].

Mechanistic in vitro evidence strongly corroborates these findings. In mature rat serotonergic neuronal cells (RN46A-B14), Sabir et al. showed that 10 nM 1,25-dihydroxyvitamin D (1,25D) normally induced tryptophan hydroxylase 1 (TPH1) and tryptophan hydroxylase 2 (*TPH2*) mRNA by 28–33-fold, and inhibited SERT and *MAO-A* mRNA by 51–59% [27]. Importantly, direct determination of serotonin levels in cell culture revealed that after 1,25D treatment, serotonin concentration had increased by 2.9. Those results prove that vitamin D is a good mimic of the joint effects of selective serotonin reuptake inhibitors (SSRIs) and MAO inhibitors at a molecular-genomic level, and that is therefore an effective explanation of the mechanistic basis of its efficacy in stabilizing moods. In addition, Patrick and Ames suggested that vitamin D, along with omega-3 fatty acids, could regulate brain serotonin by transcriptional activation of *TPH2*. Inadequate vitamin D, which afflicts about 70 percent of the world population in this model, leads to inadequate brain serotonin production, which may lead to neuropsychiatric disorders and depression [28]. This is more applicable during the postpartum period, where the steep drop in estrogen further impairs the production capacity of serotonin to aggravate the neurochemical susceptibility in relation to vitamin D deficiency.

Another interesting fact is that melatonin is also synthesized as a result of N-acetylation of serotonin in the pineal gland. Vitamin D has been implicated in circadian control; the plasma 1,25(OH)_2_D_3_ and VDBP concentrations have been shown to exhibit circadian oscillatory patterns, and *VDR* has been reported to be expressed in sleep-regulatory brain regions [2]. Deficiency of vitamin D has been associated with poor sleep quality and reduced sleep duration, which might be due to its effects on the serotonin-melatonin pathway [2]. Sleep disturbance is not only endemic in the postpartum environment, but it is also a risk factor of PPD, and the chronobiological actions of vitamin D may be another mechanism of pertinence.

#### 3.1.2. Brain-Derived Neurotrophic Factor and Neuroplasticity

Evidence presented in this section for *BDNF*-vitamin D interactions is drawn from animal models, post-stroke depression studies, and general depression populations. While these pathways are biologically plausible in PPD, extrapolation to postpartum populations should be made cautiously.

The neuroplasticity theory of depression holds that depressed states, at least partially, result from the impaired survival of neurons, the decrease in neurogenesis, and a decrease in the plasticity of synapses, especially in the hippocampus and prefrontal cortex [3,4,6,7]. The most important molecular mediator of these processes is brain-derived neurotrophic factor (*BDNF*), which facilitates neuronal growth, differentiation, and survival. A decrease in *BDNF* levels and shrinkage of hippocampal volumes have been repeatedly reported in MDD and are believed to indicate the neurobiological cost of long-term stress and inflammatory damage [6,7].

Vitamin D is associated with increased transcription of the *BDNF* gene via *VDR* transcriptional pathways, which gives a direct connection between vitamin D status and the neuroplasticity system that is involved in depression [3]. In addition to *BDNF*, vitamin D regulates a variety of other neurotrophic factors that are involved in neuronal health: it increases nerve growth factor (NGF), glial cell line-derived neurotrophic factor (GDNF), and neurotrophin-3 (NT-3). Also, it suppresses the activity of neurotrophin-4 (NT-4) [7]. Such an integrated neurotrophic assistance is congruent with the finding that sufficient vitamin D levels at key neurodevelopmental stages may have long-term effects on the emotional brain systems [28]. The neurotrophic impacts of vitamin D during the perinatal period, where the maternal brain undergoes considerable structural remodeling due to hormonal and experience influences, can have particularly important consequences on peripartum depression resilience.

Skoczek-Rubiak et al. conducted a structured narrative review that synthesized 13 studies about vitamin D status, *BDNF*, and mood or cognitive outcomes. The review established that 12 weeks of supplementation with at least 2000 IU/day lowered the scores of the Beck Depression Inventory (BDI) by 1.7–7.6 points and was also linked to 7-percentage point increases in *BDNF* levels [38]. Importantly, every 1 ng/mL increase in 25(OH)D was linked to a reduced risk of depressive symptoms, and, most notably, when levels of *BDNF* are also raised, it appears that there is an interaction between vitamin D and neurotrophic support in regulating mood. Vitamin D supplementation enhanced *BDNF* expression in the hippocampus of animal models and restored depressive behavioral losses caused by stress, showing dependency on dose across studies [38].

The direct relationship between vitamin D, inflammation, and *BDNF* through a mechanistic pathway has been shown in the context of post-stroke depression. Huang et al. observed that Vitamin D deficiency enhanced the risk of post stroke depression (PSD) by a chain-mediated mechanism, which led to an increase in IL-6 with the consequential depletion in *BDNF* (chain-mediated effect = 0.004, 95% CI 0.001–0.011, *p* = 0.032) [29]. Vitamin D supplement of PSD model rats inverted this cascade, decreasing the level of IL-6, reinstating the expression of *BDNF*, and improving the depressive-like behaviors on a sucrose preference test and forced swim test. Such a pathway, IL-6-*BDNF* decrease, is the inflammatory cascade of PPD pathophysiology, in which postpartum pro-inflammatory bursts can inhibit neurotrophic support of circuits involved in mood regulation. Moreover, Alrashed et al. in a rat model of chronic unpredictable mild stress showed that vitamin D3 supplementation abated the activation of the cGAS-STING pathway—an innovative innate immune sensing pathway triggered by mitochondrial DNA stress—and at the same time, restored hippocampal *BDNF* and *VDR* expression [30]. This incriminates the cGAS-STING pathway as another previously unknown mechanistic intermediary between vitamin D deficiency, neuroinflammation, and neurotrophic deficit, providing future research opportunities.

#### 3.1.3. Neuroinflammation and Immune Regulation

Immunomodulatory evidence presented here includes both PPD-specific studies and extrapolated evidence from general population and preclinical studies.

The inflammatory dysregulation has become one of the neurobiological processes that are most strongly supported to underlie depression [2,3,4,7]. It is always reported by case–control and longitudinal studies that people with depression have high levels of pro-inflammatory cytokines such as interleukin-6 (IL-6), interleukin-1 beta (IL-1β), tumor necrosis factor-alpha (TNF-α), and C-reactive protein (CRP) [4,7]. This inflammatory load may perpetuate depressive states in several ways: pro-inflammatory cytokines induce the enzyme indoleamine 2,3-dioxygenase (IDO), diverting tryptophan metabolism to the kynurenine pathway and serotonin synthesis; they inhibit neurogenesis in the hippocampus; and they impair the integrity of the blood–brain barrier.

Vitamin D has been shown to modulate this inflammatory cascade through its immunomodulatory actions. On the cellular level, 1,25(OH)_2_D_3_ suppresses transcription factor nuclear factor kappa-B (NF-κB), decreasing the expression of downstream pro-inflammatory genes that encode cytokines, adhesion molecules, and chemokines [2,4,7]. Vitamin D further stimulates the differentiation and activation of regulatory T cells (Tregs) that are at the center of suppressing the overreacting immune actions [7]. Vitamin D immunoregulatory effects may be particularly critical during pregnancy and in the postpartum period, which is characterized by significant immune system modulation, with a pro-inflammatory state during the first and third trimesters of pregnancy, which predisposes women to PPD [10,11].

Vitamin D’s anti-inflammatory effects at the molecular level extend to gut–brain axis modulation. Preclinical studies have shown that vitamin D3 supplementation post-sleep desynchrony (chronic circadian disruption condition) rescues microbial diversity, changes gut microbiota towards eubiosis rather than dysbiosis, and alters tight junction protein expression (ZO-1, claudin) in the proximal colon, preserving intestinal barrier integrity [28]. Since gut dysbiosis-mediated systemic inflammation is increasingly being considered as a cause of depressive symptoms via vagal and immunological mechanisms, these findings support that vitamin D can act as a gut–brain axis modulator, especially during the postpartum phase when there is a dramatic change in gut microbiota composition due to hormone changes, mode of delivery, and breastfeeding habits. The *NHANES* 2021–2023 cross-sectional analysis enhances this immunomodulatory contribution of vitamin D in depression by observing the differential effect of vitamin D isoforms: increased vitamin D3 levels were also found to be associated with less depressive symptoms, but increased vitamin D2 levels were found to be associated with increased depressive symptomatology, which again indicate isoform-specific mechanisms in inflammatory and neurochemical pathways that warrant further investigation [20].

#### 3.1.4. Hypothalamic–Pituitary–Adrenal Axis Regulation

HPA axis evidence presented in this section is derived primarily from PTSD studies and animal models, as PPD-specific HPA-vitamin D studies are limited. Extrapolation to postpartum populations requires cautious interpretation.

The hypothalamic–pituitary–adrenal (HPA) axis is the main regulator of physiological and psychological responses to stress, and it works by releasing cortisol and other glucocorticoids [3,7]. The HPA axis in healthy people displays a diurnal rhythm and sensitive negative feedback, and it allows cortisol levels to come back to normal levels after achieving stressor resolution [7]. This regulatory accuracy is disturbed in people with MDD: the characteristic features are high levels of basal cortisol, a lack of diurnal cortisol fluctuations, a high cortisol response to stress, and a negative failure in the dexamethasone suppression test, which indicates the failure of the negative feedback in the hypothalamus and pituitary [7].

The HPA axis has vitamin D receptors throughout, such as the hypothalamus, the pituitary gland, and the adrenal cortex, which gives vitamin D an opportunity to play a role in stress physiology [4,7,39]. There exists preclinical and human research showing that vitamin D can potentially exert an effect to maintain the sensitivity of glucocorticoid receptors and facilitate expression of corticotropin-releasing hormone receptor subtypes that are significant in negative feedback [3,7]. The acute post-delivery drop in estrogen and progesterone in the postpartum environment has a significant influence on HPA axis reactivity, and women who enter the postpartum environment with a lack of vitamin D might be less capable of responding with effective neuroendocrine stress responses, which may predispose them to PPD.

New clinical findings support this course. A post-traumatic stress disorder (PTSD) study demonstrated a significant decrease in the levels of serum 25-hydroxyvitamin D (*p* < 0.001) in the PTSD study group when compared to the control group. In contrast, corticotropin-releasing hormone (CRH) and adrenocorticotropic hormone (ACTH) were significantly higher. The correlation analysis of the vitamin D levels with the CRH and ACTH and the levels of cortisol showed that vitamin D deficiency is linked to the dysregulation of the HPA axis (Spearman correlation analysis: *p* < 0.05) [38]. Whereas PTSD is differentiated, PPD shares traumatic stress, HPA axis sensitization, and inflammatory sensitization, and these data have a mechanistic basis, suggesting that vitamin D status moderates neuroendocrine reactions to stress. Wu et al. also proved in animal studies that in sleep-desynchronized mice, the treatment with vitamin D3 was able to restore the function of the HPA axis and promote the suppression of corticosterone in response to the injection of dexamethasone, which further confirmed the role of vitamin D in regulating the glucocorticoid environment [29]. The sleeping impairment, combined with HPA axis malfunction, also defines the postpartum period; the dual effect of vitamin D on these systems on PPD pathophysiology is especially relevant.

#### 3.1.5. Oxidative Stress and Antioxidant Defense

The issue of oxidative stress, which is a disproportion between the formation of reactive oxygen species (ROS) and the ability to eliminate them, is becoming more and more accepted as a cause of the neurobiological cascade of depression [2,4,7]. Mitochondrial dysfunction, which is a frequent aspect of MDD, results in the overproduction of ROS, which subsequently triggers NF-κB production of pro-inflammatory cytokines, creating a vicious cycle of oxidative and inflammatory damage [2]. This cycle has the potential to interfere with *BDNF* signaling, affect serotonin metabolism, and also lead to neuronal death in mood-regulating regions of the brain.

Vitamin D has been associated with reduced oxidative stress through various mechanisms. For example, it increases the production of glutathione, the major antioxidant in the brain, by controlling gamma-glutamyl transpeptidase and promoting the expression of glutathione-forming enzymes [7]. Vitamin D also stimulates the SIRT-1 and AMPK signaling pathways and suppresses the mechanistic target of rapamycin (mTOR) and inducible nitric oxide synthase (iNOS) pathways that converge on mitochondrial integrity as well as oxidative stress [2]. Intersecting evidence also indicates that vitamin D and melatonin have common antioxidant signaling responses, which supports the fact that vitamin D status, circadian rhythm integrity, and oxidative balance are associated with depression [2]. The latter antioxidant effects could be especially useful in the postpartum period, when the metabolic needs are high, and the oxidative burden could be enhanced by the physiological stress related to delivery.

Ansari et al. also reported direct clinical evidence of antioxidant effects of vitamin D in a six-month interventional study of Arab subjects with prediabetes and vitamin D deficiency. Supplementation with vitamin D greatly enhanced the glutathione peroxidase-1 (GPx1) levels in contrast to controls (*p* < 0.01), where the levels ranged between 17.3 and 26.7 nmol/L in the intervention group [33]. One of its key enzymatic defenses against neuronal injury caused by hydrogen peroxide and lipid hydroperoxide, GPx1, has been shown to decrease in depression. Vitamin D supplementation of GPx1, therefore, has a direct mechanistic association between vitamin D status and oxidative neuroprotection of mood-regulating circuits. Moreover, Wimalawansa thoroughly explained the effects of vitamin D as a powerful antioxidant based on its capability to regulate balanced mitochondrial functions, inhibition of oxidative stress-linked protein oxidation, lipid peroxidation, and DNA damage, and epigenetic regulation by altering DNA methylation and histone modification [31]. Specifically in PPD, epigenetic mechanisms are of specific interest, as epigenetic alterations, which could be triggered by perinatal stress and nutritional deficiency, may gradually influence the expression of genes regulating mood during the postpartum period and in follow-up pregnancies.

#### 3.1.6. Calcium-Mediated Neuronal Signaling

The new explanatory mechanistic route between vitamin D and depression is the interplay in the regulation of intracellular calcium (Ca^2+^) signaling of neurons. Berridge hypothesized that depression is caused in part by the augmentation of intracellular Ca^2+^ in inhibitory GABAergic neurons, which are triggered by excessive Ca^2+^ inflow through NMDA receptors and the phosphoinositide signaling pathway, producing inositol trisphosphate (InsP3) [22]. This Ca^2+^ imbalance interferes with the equilibrium between the excitatory effects of glutamate and GABAergic inhibition to form the neural network that is linked with depressive moods. It is also worth noting that this mechanism might explain the epidemiological correlation between depression and the risk of developing Alzheimer’s disease as well, since increased Ca^2+^ signaling in the neurons might prime the production of amyloid-beta (Aβ).

Vitamin D antagonizes this pathway via what Berridge calls the ‘phenotypic stability hypothesis’: vitamin D helps to prevent the pathological accumulation of intracellular Ca^2+^ to induce neuronal dysfunction by stabilizing the expression of Ca^2+^/ATPase pumps (including plasma membrane Ca^2+^/ATPase) and Ca^2+^/buffering proteins such as calbindin-D28k, specifically, is very active in hippocampal and cortical interneurons, whose down-regulation during vitamin D deficiency may release the circuits regulating the glutamatergic release, which is also one of the reasons of hyperactivity of the stress response systems. During postpartum, estrogen tends to increase neuronal calcium buffering, and the sudden postpartum loss of estrogen can potentially expose a pre-existing calcium dysregulation predisposition in women with a concomitant vitamin D deficiency, compounding neurochemical risk factors for PPD.

### 3.2. Vitamin D and Postpartum Depression

The postpartum period is a biologically unique period of vulnerability, where hormonal, immunological, and psychosocial interplay increases the risk of mood disorders [1]. The excessive and abrupt estrogen and progesterone drop after birth, together with the initiation of stress-response mechanisms and the postpartum re-tuning of immune functions, sets the stage where nutritional determinants like vitamin D can have an outsize effect on the mental health outcomes [1,10,11]. The subsequent sections summarize the evidence on inflammatory biomarkers, systematic reviews, observational cohort studies, and trials of interventions regarding vitamin D and PPD in particular.

#### 3.2.1. Inflammatory Biomarkers and Vitamin D in the Perinatal Period

Future research has already started explaining how the interaction between inflammatory biomarkers, vitamin D status, and peripartum depressive symptoms works. Nassr et al. (in a prospective observational study) recruited a total of 80 pregnant women in Baghdad, Iraq, in the study where serum IL-6, CRP, and 25(OH)D were measured alongside depressive symptoms measured using the Edinburgh Postnatal Depression Scale (EPDS) at the third trimester and six months after delivery [11]. Association patterns were found to differ distinctly over time. Antenatal EPDS scores were also statistically significantly related to IL-6 concentrations (B = −0.025, *p* = 0.040), indicating that high levels of inflammatory signaling were negatively related to depressive symptom severity at this point [11]. Nonetheless, CRP and vitamin D levels did not show any significant direct relationships with antenatal EPDS scores in this cohort.

A different inflammatory profile was obtained during the postpartum assessment. Six months after delivery, depressive symptoms severity tended to be positively correlated with increases in CRP concentration (*p* = 0.065), indicating that greater systemic inflammation was more likely to be associated with greater severity of postpartum depressive symptoms. However, this association was not statistically significant, probably because of the small sample size in the study [11]. Notably, a significant association between CRP and serum 25(OH)D (*p* = 0.041) was observed, indicating that vitamin D status and inflammatory activity are significantly interrelated during the postpartum period [11]. This result is consistent with the known immunomodulatory properties of vitamin D and supports the hypothesis that adequate vitamin D status may be associated with reduced inflammatory burden during the postpartum period, potentially representing a modifiable factor in PPD risk.

One of the most interesting findings of the prospective study by Nassr et al. [11] was the extremely high predictive validity of antepartum depressive symptoms on postpartum outcomes: antenatal EPDS scores were significant predictors of postpartum EPDS scores (*p* < 0.001, B = 0.180, R^2^ = 0.976), which showed that depression during late pregnancy is a strong predictor of the severity of PPD. This continuity highlights the clinical importance of early prenatal diagnosis and the potential importance of prenatal risk factors like vitamin D status and inflammatory load being considered prenatally, as compared to postpartum symptoms being observed.

These results are supplemented with previous studies by Banerjee et al., who tested the effects of pro-inflammatory cytokines (IL-6 and TNF-α) and 25(OH)D in patients with Alzheimer’s disease who were depressed or not [3]. In that case–control study, participants who had concurrent depression exhibited significantly greater levels of IL-6 and TNF-alpha and lower levels of vitamin D as compared to non-depressed participants, and levels of inflammatory markers were also found to be related to disease severity [3]. Although this is not a study that was conducted postpartum, this study contributes to the overall mechanistic model where vitamin D deficiency and high levels of pro-inflammatory cytokines interact to exacerbate depressive symptoms.

The connection between inflammation, vitamin D, and depression is also supported by the general population observational data. An examination of Pakistani subjects (100 depressed and 100 healthy controls) revealed that the prevalence of vitamin D deficiency among the depressed patients was significantly high, and it was more excessive in the female group, who experience more socially related restrictions to sunshine exposure in some cultural backgrounds [34]. Akin to this, Akpinit and Karadae detected evidence of connections between low vitamin D and augmented oxidative stress and inflammation as common pathological pathways in both depression and anxiety, which concludes that vitamin D has antioxidant effects in the brain tissue that are critical to preventing and treating mood disorders [40]. Taken together, this set of results supports the multi-pathway multi-prong inflammatory-antioxidant hypothesis via which vitamin D deficiency facilitates vulnerability to depression during the perinatal stage.

#### 3.2.2. Systematic Reviews and Meta-Analyses

Several systematic reviews and meta-analyses have combined the observational and interventional evidence about maternal vitamin D status and PPD, and this is the best level of evidence currently available on this topic [3,12,13,14].

A systematic review and meta-analysis study by Yuan et al. (2024), which includes 13 studies, revealed that the levels of serum vitamin D in the depression group were significantly lower compared with those of the non-depressed controls at both prenatal and postpartum stages [14]. In the case of PPD, the pooled standardized mean difference was −1.62 (95% CI: −2.62 to −0.62), which showed that the vitamin D levels in women with PPD were significantly lower than in controls [13]. Although the heterogeneity between the studies has been high (I^2^ = 96%), the fact that the direction of the relationship has been similar in different populations justifies the existence of a significant relationship.

In a dose–response meta-analysis that involved 12 observational studies in 10,317 pregnant women, Tan et al. observed an inverse, non-linear relationship between serum levels of 25(OH)D and maternal depression risk (*p* for non-linearity = 0.001) [12]. The optimal serum 25(OH)D range for depression prevention was 90–110 nmol/L, with a pooled odds ratio of 0.49 (95% CI 0.35–0.63) comparing the highest versus lowest vitamin D levels [12]. There was a notable seasonal interaction, and more powerful protective associations were observed in summer (OR 0.25, 95% CI 0.08–0.43) compared to other seasons [12]. This seasonal restraint concurs with the reality that the production of vitamin D is not independent of the amount of sunlight, and it is biologically feasible that the association exists.

Compared to evidence of intervention, a systematic review by Gould et al. found only two RCTs of vitamin D supplementation to treat or prevent depressive symptoms during the perinatal period [11]. The two trials found that the depressive symptoms improved in the vitamin D group, although the sample sizes were too small to make concrete conclusions on the effect [11]. The 18 observational studies also included in the review had mixed results that were, to a large extent, caused by the difference in the measurement of vitamin D and depression tools, the time of measurement, and the adjustment of the results to confounders [11]. The authors assumed that the existing evidence was inconclusive and that prospective research should include validated measures of depression as pre-specified outcomes in large prenatal vitamin D studies [11].

A larger umbrella meta-analysis synthesized 10 RCT meta-analyses and 4 cohort meta-analyses by Musazadeh et al. reported that vitamin D supplementation significantly lowered depressive symptom scores with a pooled standardized mean difference (SMD) −0.40 (95% CI −0.60–−0.21), whereas inadequate vitamin D status was linked to a 60% higher odds of depression (pooled OR 1.60) in observational analyses [3]. Recently, a meta-analysis published in 2025 by Wang et al. used 20 RCTs published between January 2000 and October 2024 and found a moderate but statistically significant antidepressant effect of vitamin D supplementation (SMD = −0.36; 95% CI −0.52 to −0.20; *p* < 0.00001), reinforcing the clinical relevance of addressing vitamin D deficiency in depression management [41]. This meta-analysis was general and not postpartum-specific, and the effect size is in line with previous pooled estimates and provides the biological plausibility of PPD-focused interventions.

A selective systematic review performed by Rosian et al. involving 70 articles analyzed 13,976 studies, with cross-sectional, cohort, and interventional study designs, showed the inverse relationship between serum 25(OH)D and the risk and severity of depression and concluded vitamin D as a potential adjunctive therapy treatment factor of MDD [8]. Directionality was positive, although inconsistent effects of vitamin D supplementation on depression were also reported in a narrative review by Bersani et al., and the most consistent evidence regarding vitamin D supplementation was noted in populations with pre-existing vitamin D deficiency [7]. The narrative review of Kunugi further contextualized vitamin D in the context of a broader lifestyle medicine model, with nutritional prevention of a vitamin D deficiency being a reasonable preventive measure of depressive disorders, especially where multiple lifestyle risk factors intersect [46]. All these reviews highlight the urgent necessity of sufficiently powered, postpartum-specific RCTs.

#### 3.2.3. Observational Studies: Vitamin D Status and Postpartum Depression

Substantial prospective cohort studies have provided valuable epidemiological evidence supporting an association between prenatal vitamin D status and subsequent PPD or postpartum mood symptoms [15,16,18].

Domacassé et al. based their research on the Amsterdam Born Children and their Development (ABCD) cohort of 2483 participants and measured maternal serum vitamin D at a median gestation of 13 weeks and postpartum depressive and anxiety symptoms at 3 months postpartum [14]. Vitamin D deficiency (≤29.9 nmol) after controlling for a set of confounders was linked with heightened postpartum anxiety symptoms in comparison to the normal level of vitamin D (B = 0.17, 95% CI = 0.03–0.30, *p* = 0.017) [15]. In women who did not take vitamin D supplements during pregnancy, vitamin D deficiency was associated with both increased anxiety (B = 0.17, 95% CI = 0.03–0.32, *p* = 0.015) and depressive symptoms (B = 0.14, 95% CI = 0.03–0.28, *p* = 0.045) at three months postpartum [15]. The mean vitamin D concentration in women with comorbid PPD and anxiety was found to be 51.3 nM (which is well below the ideal value), and 32.1 percent of the women had vitamin D deficiency [15]. The observation that no associations were found between these variables in the unsupplemented women indicates that prenatal vitamin D supplementation could reduce the vulnerability of women to postpartum mood, but this is subject to prospective confirmation.

Tsunoda et al. carried out the biggest study (one to date) on vitamin D and PPD in the diet of the population in Japan in the Japan Environment and Children’s Study (JECS) cohort. They included 74,840 pregnant women [16]. Pregnant women were measured on dietary vitamin D consumption using a dietary history of food frequency questionnaire. They were measured on symptoms of postpartum depression one month after giving birth using the EPDS. The dose–response pattern was significant, as women in the second through the fifth quintile of dietary vitamin D intake had significantly lower odds of PPD than those in the lowest quintile: adjusted ORs of 0.88 (95% CI 0.82–0.94), 0.83 (0.78–0.89), 0.87 (0.81–0.93), and 0.90 (0.83–0.97) respectively, with a statistically significant trend (*p* = 0.004) [16]. The result of an exceptionally large, nationally representative sample offers strong epidemiological support that dietary vitamin D in pregnancy is negatively related to the symptoms of PPD, even after the adjustment of sociodemographic and obstetric variables.

The retrospective study of village primary health care in the rural setting of Australia by Ogiji and Rich revealed that 57.1% of pregnant women were screened against vitamin D in the antenatal phase, with 66.3% screened having improper serum vitamin D content (23.9% deficient; 42.4% suboptimal) [18]. Females with PPD had a much lower level of antenatal vitamin D than the women without PPD (*p* < 0.01), and a remarkably negative correlation was noted between the level of antenatal vitamin D and PPD. This primary care study shows that non-disastrous antenatal vitamin D deficiency is widespread and clinically pertinent in affluent locations. In Iran, Abedi et al.’s case–control study of 120 women (60 with PPD, 60 controls) found that the mean vitamin D level in women with PPD was 16.89 ± 7.05 ng/mL versus 21.28 ± 7.13 ng/mL in controls (*p* = 0.001) [19]. Women with vitamin D below 20 ng/mL were 3.3 times more likely to develop PPD (OR 3.3, 95% confidence interval (CI) 1.32–8.24, *p* = 0.01). These clinical results are similar to a review by Prijadi et al., which summarized 11 studies and reported that 9 of 11 showed a significant association between vitamin D levels and PPD, with the two studies indicating insignificance varying by timing of bacteriology and the depression instrument utilized [42]. Diversity in approach—especially the choice to measure serum vitamin D or dietary intake, and the choice to measure PPD at one month versus six months postpartum—should also be the most important source of heterogeneity among the body of observational evidence.

A large Chinese retrospective study by Lv et al. offered valuable interventional data in the prenatal phase: in 1365 women diagnosed with vitamin D-deficiency during the 12–14 weeks of pregnancy, propensity-score matched analysis demonstrated that daily supplementation with 800 IU of vitamin D after 14 weeks of pregnancy markedly improved the score of prenatal depression, as compared to the unsupplemented deficiency group (*p* < 0.001) [35]. Although this was a prenatal and not a PPD study, there is evidence of a positive impact of vitamin D insufficiency (not patient deficiency) status on rates of depressive symptomology clinical change in support of a supplementation hypothesis in perinatal settings. An analogous result of the critical appraisal by Menon et al. is that supplementation trials’ evidence is most convincing in the case of subjects with major depression and cohesive vitamin D deficiency, and therefore, that targeted supplementation, or identifying and treating the deficient individuals, could produce the most significant clinical impact [43].

While the observational evidence consistently demonstrates inverse associations between vitamin D status and PPD risk, several methodological limitations warrant careful consideration before inferring causality. First, the possibility of reverse causation cannot be excluded: depressive symptoms during pregnancy may lead to reduced outdoor activity, poor dietary intake, and diminished self-care, all of which could lower vitamin D levels rather than vitamin D deficiency causing depression. Second, residual confounding is a persistent concern despite covariate adjustment; factors such as socioeconomic status, season of assessment, ethnicity, physical activity, and overall nutritional status are incompletely captured in many studies. Third, publication bias may contribute to the preponderance of positive findings, as studies reporting null or negative associations are less likely to be published. Fourth, the high statistical heterogeneity (I^2^ = 96% in Yuan et al., I^2^ = 82.1% in Tan et al.) [13] indicates substantial variation in effect sizes across studies, suggesting that the relationship may be context-dependent or influenced by unmeasured moderators. Fifth, the cross-sectional design of many included studies precludes assessment of temporal relationships. These limitations collectively suggest that while the association is robust, its causal nature and clinical significance require confirmation through well-designed intervention studies.

Put together, these prospective cohort results lead to the same direction, i.e., poorer maternal vitamin D levels during pregnancy, be it through serum levels or through food intake, are correlated with more postpartum depressive and anxiety symptoms. The future time-course, the dose–response curve in large cohorts, and the conceivable biological interactions all lend weight to the argument of a significant, but not necessarily causal, association.

#### 3.2.4. Intervention Trials

Although the observational evidence has been rather strong, the literature on interventions specific to PPD is scant. A systematic review by Gould et al. found two RCTs of vitamin D supplementation, the purpose of which was to prevent or treat postpartum depressive symptoms. However, neither was large enough to provide conclusive results [12]. Other relevant background information is found in the broader RCT literature on the use of vitamin D supplementation and depression in non-postpartum populations: meta-analyses of interventional studies have reported significant decreases in depression symptom scores with supplementation (pooled standardized mean difference: −0.40; 95% CI: −0.60, −0.21) particularly at doses of ≥800 IU/day and in individuals with baseline deficiency [3]. Nevertheless, the heterogeneity of these trials is high, as it indicates a difference in dosing, period, pre-tested vitamin D levels, and the level of depression.

When it comes to the optimal dose, the narrative review provided by Skoczek-Rubińsk et al. hints that higher dosages of at least 2000 IU/day during at least 12 weeks are required to induce clinically significant changes in the degree of depressive symptoms and large alterations in *BDNF* [38]. This amount is quite high, compared to the 400–800 IU/day commonly found in typical prenatal vitamin supplements, implying that existing routine supplementation regimens are not sufficient to treat vitamin D deficiencies of clinical severity and neurobiological outcomes. In severely deficient individuals, pharmacotherapy (50,000 IU per week over 2 months followed by maintenance) may be needed to ensure normalization of serum levels of 25(OH)D into the protective range of 90–110 nmol/L as observed in dose–response meta-analyses [13]. Pharmacokinetically informed dosing algorithms, taking into consideration background 25(OH)D, body mass index, and endogenous vitamin D production season, should be used to design future RCTs.

Vitamin D interaction with other nutritional interventions also deserves attention in designing future trials. The review by De Cillis et al. points out that the similar mechanisms of the action of omega-3 polyunsaturated fatty acids (n-3 PUFAs) and vitamin D in depression involve the regulation of inflammatory cytokines, *BDNF* pathways, HPA axis, and oxidative stress [23]. Combined nutrient deficiency is a frequent occurrence in the postpartum period—the amount of docosahexaenoic acid (DHA) decreases with each additional pregnancy, and there is a high prevalence of vitamin D deficiency in the world; this indicates that pilot trials of combination supplementation may be more effective intervention options compared to either nutrient. The multi-nutrient view of brain function and risk of depressive symptoms is further supported by the review of nutrient deficiencies by 47 studies by Zielińska et al., which found interventions targeting multiple deficiencies concurrently could also have a superior clinical effect [24]. Table 2 summarizes the key characteristics and findings of all 46 studies included in this review. Studies are organized into three categories: mechanistic studies (Table 2A), observational studies (Table 2B), and interventional/systematic review studies (Table 2C). To aid readability, the ‘Notes’ column highlights whether findings are PPD-specific or extrapolated from other populations. For detailed methodological information, readers are referred to the original publications.

## 4. Discussion

This narrative review summarizes the biological, epidemiological, and interventional literature that investigates the connection between the status of vitamin D and PPD. Combined mechanics of mechanistic plausibility, ongoing observational data, and emerging intervention data support the hypothesis that sufficient vitamin D in perinatal care might be significantly protective against postpartum depressive and anxiety symptoms, although a definite causal finding is in the early stages.

The mechanistic case of the role of vitamin D in postpartum mood regulation is the strongest aspect of the existing evidence base. As summarized in Table 3, the multi-pathway model involves serotonergic neurotransmission, neuroplasticity via *BDNF*, neuroinflammatory suppression, modulation of the HPA axis, and oxidative stress defense, each supported by preclinical and general-population studies [2,4,22,26]. Notably, these pathways have been shown to constitute a complex, synergistic structure through which the deficiency of vitamin D can simultaneously reduce serotonin synthesis, block neurotrophic support, increase inflammatory cytokine effects, and respond in a dysregulated manner to stress-axis activation. The postpartum biological data enhance the relevance of all the mechanisms: estrogen withdrawal exacerbates serotonergic vulnerability, immune activation postpartum intensifies pro-inflammatory events, and the sudden loss of neuroprotective calcium buffering by estrogen places neurons at greater risk of Ca^2+^ dysregulation, as has been described by Berridge. More recent mechanisms such as cGAS-STING innate immune activation, gut microbiota control, and epigenetic control of mood-relevant genes add another layer to this paradigm and may indicate that the postpartum period is a sensitive window during which the vitamin D status may disproportionately influence mood outcomes.

A key consideration when interpreting the vitamin D–PPD literature is the substantial disconnect between the biological plausibility of the association and the quality of the clinical evidence available to support it. The mechanistic pathways outlined in Section 3.1 encompassing serotonin synthesis, *BDNF* signaling, neuroinflammation, HPA axis regulation, and oxidative stress defense are robustly supported by preclinical and general population studies. However, these pathways have rarely been examined simultaneously in perinatal cohorts, leaving critical questions unanswered about which mechanisms are most relevant to postpartum women specifically, whether vitamin D acts through all pathways or predominantly through one, and whether the magnitude of effect through each pathway is clinically meaningful. Moreover, the postpartum period presents unique biological conditions including abrupt hormonal withdrawal, immune system shifts, and sleep disruption that may either amplify or alter the neurobiological effects of vitamin D compared to non-pregnant populations. Without mechanism-focused studies in postpartum women, the extrapolation from preclinical models and general population research, while informative, remains speculative. This evidence gap is particularly problematic because it complicates the interpretation of observational findings: if vitamin D acts through multiple pathways, then the modest effect sizes observed in some studies may reflect the sum of small effects across pathways, whereas if a single dominant mechanism exists, the observed associations should be more consistent and larger. The current evidence does not allow discrimination between these possibilities, underscoring the need for perinatal research that integrates biomarker measurement with clinical outcome assessment.

The directional consistency of the epidemiological evidence has been notable across varied settings and must be interpreted carefully. The most granular epidemiological estimate that exists at present is given by the dose–response meta-analysis of Tan et al., where the best range of serum 25(OH)D is 90–110 nmol/L with a pooled odds ratio of 0.49 [13]. The results of the Mendelian randomization study by Lyu et al., which show that there is a significant negative genetic association between the amount of vitamin D in the body and depression in the presence of shared neurodevelopmental loci, provide a causally informative complement to the usual observational techniques [21]. Future research incorporating genetically informed designs and comprehensive covariate assessment will be essential to distinguish causal effects from confounding and reverse causation.

The critical gap is the statistics of the interventions. Fewer than two small, underpowered RCTs specifically address vitamin D supplementation with regard to postpartum depressive symptoms, which reflects significant disproportions among the biological and observable signals of postpartum depressive symptoms and the available trial data. Larger RCT meta-analyses of non-targeted populations in general, such as the 20-trial study by Wang et al., which reports a pooled SMD of −0.36, help in endorsing the idea of antidepressant benefit of supplementation, but cannot be directly extended to women postpartum due to the specific characteristics of the time period. The growing recognition of the need to administer daily doses of at least 2000 IU/day during a minimum of 12 weeks to induce clinically significant changes is an extreme contrast to the dosages of 400–800 IU/day commonly administered in standard prenatal preparations, and indicates that existing regimens of prenatal supplementation might not have a full impact on maternal mental health responses [44]. The possible interaction of vitamin D and the omega-3 fatty acids—which are parallel deficient in women at the postpartum and which have common mechanistic targets such as regulation of the inflammatory cytokines and *BDNF* signaling—is also to be tested in specific combination studies [23,25].

In low- and middle-income countries, darker skin pigmentation substantially reduces cutaneous vitamin D synthesis in response to ultraviolet B radiation, a phenomenon well-documented in the literature; this biological reality creates an urgent and unmet need for vitamin D supplementation among sub-Saharan African women, who face the dual burden of high melanin content and high rates of vitamin D deficiency [36].

Future studies must focus on large, pre-registered, RCTs in postpartum cohorts using clinically adequate dosing, PPD as a prime outcome measure, and mechanistic biomarker sub-studies therein. The trials must be done in a variety of geographic locations; genetic stratification comprising variants of vitamin D metabolism should be carried out, as well as the comparative and joint effect of vitamin D3 and D2, and co-supplementation with omega-3 fatty acids should be done. Mendelian randomization designs provide an alternative path to causality. Taken together, these initiatives are required to transform a compelling observational and biological cue into clinical guidance that can be acted upon in the case of maternal mental health.

## 5. Limitations

This review has several important limitations that must be considered when interpreting the evidence:

1. **Limited PPD-specific evidence base:** The most significant limitation is the scarcity of studies directly examining vitamin D and PPD. We identified only two RCTs specifically addressing vitamin D supplementation for PPD prevention or treatment, both underpowered and methodologically heterogeneous. This represents a critical evidence gap that prevents definitive conclusions about causality, optimal dosing, timing, and clinical efficacy in postpartum populations.
2. **Small number of PPD-specific observational studies:** While observational studies consistently show inverse associations, the number of PPD-specific studies remains relatively small (only 10 observational studies included in this review). Many of these studies have modest sample sizes (e.g., n = 80 in Nassr et al., n = 120 in Abedi et al.) and may be underpowered to detect modest effects or interaction effects.
3. **Geographic limitations:** The majority of PPD-specific studies were conducted in high-income settings (Netherlands, Japan, Australia, Iran) or single-country contexts. This limits generalizability to low- and middle-income countries where vitamin D deficiency and PPD prevalence are highest and where the burden of both conditions is disproportionately borne by women.
4. **Methodological heterogeneity:** Variability in vitamin D measurement (serum 25(OH)D, dietary intake, supplementation), timing of assessment (first trimester, third trimester, postpartum), depression measurement instruments (EPDS, PHQ-9, BDI), and deficiency thresholds (varying from <50 nmol/L to <75 nmol/L) complicates cross-study comparison and meta-analytic pooling.
5. **Extrapolation from non-postpartum populations:** As discussed in Section 4, much of the mechanistic evidence is extrapolated from preclinical models and general population studies. While these provide valuable mechanistic insights, their direct applicability to postpartum populations requires confirmation through perinatal-focused research.
6. **Narrative review limitations:** This is a narrative review with systematic search, not a formal systematic review with meta-analysis. The narrative synthesis approach, while appropriate given the heterogeneity of study designs and populations, may be subject to interpretive bias and does not provide quantitative pooled effect estimates.
7. **Publication bias:** The predominance of positive findings in the published literature may reflect publication bias, with studies reporting null or negative associations being less likely to be published.
8. **Insufficient mechanistic sub-studies:** Few studies have simultaneously measured vitamin D, inflammatory biomarkers, *BDNF*, and depressive symptoms in perinatal populations, limiting understanding of the mechanistic pathways in the postpartum context.

## 6. Conclusions

This narrative review is an overview of the evidence that vitamin D status can correlate with PPD. All the biological mechanistic data, the large-scale prospective epidemiological studies, and the meta-analytic data lead to the conclusion that being in an adequate state of vitamin D during pregnancy and the postpartum period is associated with a reduced risk and severity of postpartum symptoms of depression and anxiety. The biological plausibility of such a relationship is established: vitamin D regulates the production and metabolism of serotonin, increases neuroplasticity through *BDNF* and other neurotrophic factors, suppresses the pro-inflammatory cytokine cascade of depression, modulates the responsiveness of the HPA axis, and decreases oxidative stress in brain regions of mood mediation. These mechanisms are not mutually exclusive, but rather interact in an integrated and mutually reinforcing way that predisposes vitamin D to be quite a versatile candidate in the neurobiology of postpartum mood disorders [32,44,45]. Also, more recently described interventions of calcium-mediated neuronal signaling, cGAS-STING activation of innate immune pathways, and the effects of the gut–brain axis and the epigenomic regulation of the expression of mood-relevant genes continue to enrich the mechanistic models in which vitamin D deficiency can further intensify postpartum depressive susceptibility.

The observational evidence is quite consistent. Potential cohort studies in a variety of geographic and cultural settings—the Netherlands to Japan to Iraq—describe the presence of inverse relationships between maternal vitamin D levels and postpartum depressive and anxiety symptoms. Dose–Response meta-analyses indicate that the most desirable serum 25(OH)D levels were 90–110 nmol/L, with the lowest risk of PPD, and that large cohort studies show statistically significant decreases in the risk of PPD with increasing quintiles of dietary vitamin D intake. Women who are deficient in vitamin D in the first trimester, especially those not receiving vitamin D supplements, seem to be at high risk of both depressive and anxiety symptoms in the postpartum period, which indicates that the critical period in which to intervene could be during early pregnancy or even preconceptionally. The case–control data in Iran and Australia indicate identical strengths of association with a setting under the 20 ng/mL cut-off, showing that women who surpassed this cut-off had a threefold higher odds of PPD.

The existing evidence base cannot be utilized to make clinical recommendations regarding vitamin D supplementation as a specific intervention to prevent PPD. This question is only discussed in postpartum populations in two small and methodologically heterogeneous RCTs, and causality has not been proven. Sufficient vitamin D in pregnancy is also a logical aspect of overall maternal nutrition education—in line with respectable advice on bone health and fetal development—but should not as yet be put among all these used to establish vitamin D supplementation as a method of preventing PPD. Vitamin D deficiency screening could be an option in women with known risk factors (minimal sun exposure, dark skin pigmentation, poor nutritional intake), based on the concept of overall nutritional health, as opposed to targeted PPD prevention. Given the safety profile and low cost of vitamin D3 supplementation (2000 IU/day), some would argue that empirical supplementation in high-risk groups may be preferable to the relatively expensive laboratory testing required for screening.

Future studies should focus on large, well-designed RCTs with PPD as a pre-specified first or second outcome, recruiting women with proven vitamin D deficiency, using clinically significant doses (at minimum 2000 IU/day based on emerging evidence, and extending the follow-up to the entire first year of postpartum. These trials should be supplemented by mechanistic sub-studies of biomarkers of serotonin metabolism, inflammatory cytokines, *BDNF*, and the HPA axis to contribute significantly to the knowledge of the involved pathways. Mendelian randomization designs that use genetic variants related to vitamin D metabolism provide a complementary method to causal inference and do not have the confounding of observational designs. Trials in populations other than high-income Western countries are also needed, as both vitamin D deficiency and PPD are a global burden. The groups with the highest prevalence of the former are demographic groups. Since there is overlapping genetic architecture in vitamin D levels and depression, as demonstrated in GWAS, genetic stratification in future trials can allow identification of subpopulations with the greatest likelihood of benefiting from the supplement. In addition, the combination of vitamin D and omega-3 supplementation with fatty acids should be the subject of trials, as the two mechanisms overlap and are usually co-deficient in postpartum women. Research must also be conducted in low- and middle-income country settings—where both shortages are more prevalent—to look at the global equity aspects of postpartum mental health.

In conclusion, the current evidence has presented a strong biological and epidemiological argument that vitamin D is a nutritional factor that can be applied to postpartum mental health. To translate this observational signal into clinical practice, it will be necessary to produce strong interventional evidence. As a short-term measure, vitamin D deficiency as a component of holistic perinatal care is a reasonable and effective public health intervention.

## Figures and Tables

**Table 1:** Inclusion and exclusion criteria for the literature search of the studies included in this narrative review evaluating the association between vitamin D status and postpartum depression (PPD).

Inclusion Criteria	Exclusion Criteria
Studies published 2015–2026	Studies focused exclusively on antenatal depression without postpartum follow-up
Vitamin D status, dietary intake, or supplementation as exposure	Conference abstracts and dissertations
Depression or PPD as primary outcome	Studies without validated depression assessment tools
Randomized controlled trials, cohort, case–control, cross-sectional studies	Studies with no measure of vitamin D status
Systematic reviews and meta-analyses	
Full-text available in English	

**Table 2:** Summary of mechanistic, systematic review, observational, and intervention studies evaluating the relationship between vitamin D status or supplementation and postpartum depression (PPD).

**(A): Mechanistic Studies**					
**Study**	**Category**	**Design**	**Sample**	**Key Findings**	**Notes**
[2]	Mechanistic/Biological	Perspective review	—	Helps brain produce good chemicals	Lab evidence only
[6]	Mechanistic/Biological	Observational (genome-wide association study (GWAS)+ cohort); UK Biobank	Large cohort	Lower vitamin D increasing the risk of depression in general adults	Findings from general adults-not new mothers.
[10]	Mechanistic/Biological	Case–control	n = 120 (60 controls, 26 AD without depression, 34 AD with depression)	Inflammation and Low vitamin D linked to worse mood	From dementia patients, mechanism may apply but not proven in PPD
[21]	Mechanistic/Biological	GWAS + Mendelian randomization	VD N = 417,580; Depression N = 589,356	Genes suggest vitamin D directly affects depression risk	Strong genetic evidence, but not from mothers
[22]	Mechanistic/Biological	Review of cellular mechanisms	—	Vitamin D protects brain cells from calcium overload	Lab-based theory— needs PPD confirmation
[23]	Mechanistic/Biological	SR of biological pathways	Narrative SR	Vitamin D and fish oil work together to protect the brain.	Combined nutrient approach may be more effective
[25]	Mechanistic/Biological	Animal model (sleep desynchrony)	C57BL/6J mice	Vitamin D helps restore normal stress response after sleep loss.	Relevant to postpartum sleep deprivation-needs testing.
[26]	Mechanistic/Biological	Molecular review	Preclinical + clinical	Vitamin D protects the brain through multiple pathways.	Strong biological case-more PPD research needed.
[27]	Mechanistic/Biological	In vitro (rat serotonergic cells)	RN46A-B14 cells	Vitamin D directly boosts serotonin in brain cells.	Strong lab evidence-needs human confirmation.
[28]	Mechanistic/Biological	Mechanistic review	—	Most people lack enough vitamin D for optimal brain serotonin.	Foundational study-widely cited for serotonin-vitamin D link.
[29]	Mechanistic/Biological	Clinical + animal model	390 patients + rats	Vitamin D breaks the “inflammation, brain damage, depression” chain.	Same inflammation pathway may apply to postpartum.
[30]	Mechanistic/Biological	Animal model (CUMS rats)	32 rats	Vitamin D calms brain inflammation in stressed animals.	New mechanism discovered-needs PPD research.
[31]	Mechanistic/Biological	Review of molecular mechanisms	—	Vitamin D acts like a “master switch” for brain cell health.	Broad mechanism review-lays foundation for PPD research.
[32]	Mechanistic/Biological	Review of preclinical evidence	Rodent models	Vitamin D acts like a natural antidepressant in animal studies.	Latest lab evidence-translation to humans needed.
**(B): Observational Studies**					
**Study**	**Category**	**Design**	**Sample**	**Key Findings**	**Notes**
[1]	Observational	Meta-analysis of prevalence studies	412 studies; n = 792,055 women	PPD affects 1 in 5 mothers globally.	PPD research is mandatory
[5]	Observational	Systematic review	95 studies	Most pregnant women worldwide have low vitamin D.	Shows vitamin D deficiency is a global problem.
[11]	Observational	Prospective observational	n = 80	Depression in pregnancy is the strongest PPD predictor.	Small study-results need replication.
[16]	Observational	Prospective cohort	n = 74,840	Consuming more vitamin D during pregnancy lowers PPD risk.	Largest study to date-very strong evidence.
[18]	Observational	Retrospective observational	Rural Australian practice	Low vitamin D is common and linked to PPD in rural Australia.	Real-world data but cannot prove cause and effect.
[19]	Observational	Case–control	n = 120	Very low vitamin D = 3-fold higher PPD risk.	Strong association from case–control study.
[33]	Observational/Interventional	Non-randomized intervention	n = 203	Vitamin D boosts brain-protecting antioxidants.	Shows antioxidant mechanism—relevant to PPD.
[34]	Observational	Case–control	200 subjects (100 depressed, 100 healthy)	Depression linked to low vitamin D-women most affected.	Cultural factors affect vitamin D in women.
[35]	Observational/Interventional	Retrospective; propensity-score matched	n = 1365	Even moderate vitamin D supplementation reduces depression in pregnancy.	Supports supplementation but outcome was prenatal, not postpartum.
[36]	Observational	Observational clinical study	96 PTSD patients	Low vitamin D = abnormal stress response in trauma patients.	Stress mechanism may apply to postpartum.
**(C): Interventional and Review Studies (Systematic Reviews and Meta-Analyses)**					
**Study**	**Category**	**Design**	**Sample**	**Key Findings**	**Notes**
[3]	Systematic Review/Meta-Analysis	Umbrella meta-analysis (interventional + observational MAs)	10 RCT meta-analyses + 4 cohort meta-analyses	Vitamin D helps depression generally but not yet proven for PPD.	General population data promising-PPD trials needed.
[4]	SR/Meta-Analysis	Review of hypotheses and mechanisms	Literature review	Multiple pathways connect vitamin D to mood.	Good background reading for mechanistic understanding.
[7]	Systematic Review/Meta-Analysis	Narrative review of prospective & RCT literature	Prospective longitudinal studies + RCTs	Link exists, but supplement results are mixed.	Mixed results-highlights need for better studies.
[8]	Systematic Review/Meta-Analysis	Systematic review (cross-sectional, cohort, RCTs)	70 articles (from 13,976 screened)	Vitamin D may help as add-on treatment for depression.	Adjunctive role suggested-needs PPD confirmation.
[9]	SR/Meta-Analysis	SR (PubMed, Scopus, WoS)	Multiple studies	Evidence supports checking vitamin D in pregnancy.	Latest PPD review-screening recommended.
[13]	Systematic Review/Meta-Analysis	Dose–response meta-analysis	12 observational studies; n = 10,317	Optimal vitamin D halves PPD risk—target is 90–110 nmol/L.	Strong dose–response evidence but not from RCTs.
[14]	Systematic Review/Meta-Analysis	Systematic review & meta-analysis	13 studies	Women with PPD clearly have lower vitamin D levels.	Consistent finding, but studies vary widely.
[17]	SR/Meta-Analysis	Narrative review	Multiple studies	Biology is clear—clinical evidence is not yet.	Strong mechanistic case—weak clinical proof.
[24]	SR/Meta-Analysis	Review 2018–2023	Multiple studies	One deficiency is bad; multiple deficiencies are worse for mental health.	PPD may benefit from correcting multiple deficiencies.
[37]	SR/Meta-Analysis	Review (cognitive/neuroprotective)	Multiple studies	Vitamin D broadly protects the aging brain.	Brain protection applies across conditions.
[38]	SR/Meta-Analysis	Structured narrative review	13 studies	Need at least 2000 IU daily for 3 months to see mood improvement.	Useful dosing information-higher than prenatal vitamins.
[39]	SR/Meta-Analysis	Review of HPA mechanisms	Multiple studies	Stress damages mood-regulating brain areas.	Stress mechanisms relevant to postpartum period.
[40]	SR/Meta-Analysis	Narrative review	Multiple studies	Low vitamin D increases both depression and anxiety via inflammation.	Mechanism applies to both conditions.
[41]	SR/Meta-Analysis	Meta-analysis of 20 RCTs	20 RCTs	Large meta-analysis confirms vitamin D helps depression-modest but real effect.	Strongest RCT evidence-but general population.
[42]	SR/Meta-Analysis	Literature review	11 studies	Most studies (9 of 11) link low vitamin D to PPD.	Timing and measurement methods matter greatly.
[43]	SR/Meta-Analysis	Narrative review	61 articles	Vitamin D helps most in those who are truly deficient.	Test first, then treat-do not assume it works for everyone.
[44]	Systematic Review/Meta-Analysis	Protocol for systematic review & meta-analysis	RCTs (perimenopausal women)	Protocol only—no results yet.	Methodological example—no evidence for PPD.
[45]	SR/Meta-Analysis	Narrative review	Multiple studies	Vitamin D affects both serotonin and inflammation-key to mood and suicide prevention.	Broader public health relevance.

(**A**) Abbreviations: AD, Alzheimer’s disease; GWAS, genome-wide association study; PPD, postpartum depression; UK, United Kingdom; VD, Vitamin D. (**B**) Abbreviations: ABCD, Amsterdam Born Children and their Development; PPD, postpartum depression; PTSD, Post-traumatic stress disorder; VD3, Vitamin D3; HPA, Hypothalamic–Pituitary–Adrenal Axis. (**C**) Abbreviations: CI, confidence interval; OR, odds ratio; PPD, postpartum depression; RCT, randomized controlled trial; SMD, standardized mean difference; VD, vitamin D. SR = Systematic Review; MA = Meta-Analysis; GWAS = Genome-Wide Association Study; CUMS = Chronic Unpredictable Mild Stress; PTSD = Post-Traumatic Stress Disorder. Summary of Table 2: Across all 46 studies, observational evidence consistently demonstrates inverse associations between vitamin D status and PPD (Table 2B). However, intervention evidence remains limited, with only two small RCTs identified (Table 2C). Mechanistic studies (Table 2A) provide biological plausibility but are predominantly derived from non-postpartum populations.

**Table 3:** Proposed biological mechanisms linking vitamin D status with postpartum depression (PPD), including neurotransmitter regulation, inflammation, oxidative stress, HPA-axis function, and calcium signaling.

Mechanism	Role of Vitamin D	Implication for Depression
Neuroplasticity/*BDNF*	VD upregulates *BDNF*, NGF, GDNF and *NT-3* gene expression via *VDR*-mediated transcription	Reduced neurogenesis and hippocampal volume linked to major depressive disorder (MDD); VD insufficiency may exacerbate hippocampal atrophy [4,7]
Neuroinflammation	VD inhibits NF-κB, suppresses IL-6, IL-1β, TNF-α, and promotes regulatory T-cell differentiation [2,4,7]	Pro-inflammatory cytokines activate IDO, shunting tryptophan toward kynurenine rather than serotonin
HPA axis regulation	*VDR* present in hypothalamus, pituitary, and adrenal cortex; VD modulates glucocorticoid responses [2,7]	Dysregulated HPA axis (elevated cortisol, impaired negative feedback) is a hallmark of MDD
Oxidative stress and mitochondria	VD upregulates glutathione synthesis, sirtuin-1 (SIRT-1), and AMP-activated protein kinase (AMPK) pathways; downregulates mTOR and iNOS	Oxidative stress disrupts *BDNF* signaling and amplifies neuroinflammation in a self-reinforcing cycle [2,7]
Chronobiology and melatonin	VD influences circadian oscillation of 1,25(OH)_2_D_3_ and DBP; *VDR* expressed in sleep-regulatory brain areas [2,4,7]	VD deficiency linked to impaired sleep quality and disrupted melatonin production
Calcium-mediated neuronal signaling	VD maintains expression of Ca^2+^ pumps and buffers; reduces NMDA receptor-mediated Ca^2+^ overload in inhibitory neurons	Excessive intracellular Ca^2+^ in GABAergic neurons contributes to glutamate-excitatory imbalance and depressive states; may link depression and Alzheimer’s risk [22]
Serotonergic pathway	1,25(OH)_2_D_3_ upregulates *TPH2* gene expression, modulates SERT activity, and reduces *MAO-A* activity [2,7]	Low VD impairs serotonin synthesis and elevates degradation, contributing to mood dysregulation [27]
Gut microbiota/gut–brain axis	VD3 restores gut microbial diversity after dysbiosis; upregulates tight junction proteins (ZO-1, claudin) to maintain intestinal barrier integrity	Gut dysbiosis and increased intestinal permeability drive systemic inflammation and vagal signaling disruption associated with depression [28]
Glutathione peroxidase/antioxidant enzyme upregulation	VD supplementation significantly increases glutathione peroxidase-1 (GPx1) levels in deficient populations	Enhanced GPx1 activity reduces neuronal oxidative damage implicated in depressive neurodegeneration
cGAS-STING neuroinflammatory pathway	VD3 attenuates cGAS-STING signaling in hippocampus under chronic stress; reduces microglial activation (Iba1) [32]	cGAS-STING activation links innate immune sensing to neuroinflammation and *BDNF* suppression in stress-induced depression
Epigenetic and gene regulatory effects	VD influences DNA methylation, histone modification, and expression of >200 genes via *VDR*-mediated transcription and vitamin D response elements (VDREs) [40]	Epigenetic dysregulation may perpetuate depressive phenotypes across the lifespan; VD may reset gene expression patterns relevant to mood

**Abbreviations:** adrenocorticotropic hormone; AMPK, AMP-activated protein kinase; *BDNF*, brain-derived neurotrophic factor; Ca^2+^, calcium ion; GABA, gamma-aminobutyric acid; GPx1, glutathione peroxidase-1; HPA, hypothalamic–pituitary–adrenal; IDO, indoleamine 2,3-dioxygenase; IL-6, interleukin-6; iNOS, inducible nitric oxide synthase; *MAO-A*, monoamine oxidase A; mTOR, mechanistic target of rapamycin; NF-κB, nuclear factor kappa B; NMDA, N-methyl-D-aspartate; PPD, postpartum depression; SERT, serotonin transporter; TNF-α, tumor necrosis factor-alpha; *TPH2*, tryptophan hydroxylase 2; VD, vitamin D.

## Data Availability

No new data were created or analyzed in this study. Data Sharing is not applicable to this article.
